# SLE: a cognitive step forward—a synthesis of rethinking theories, causality, and ignored DNA structures

**DOI:** 10.3389/fimmu.2024.1393814

**Published:** 2024-06-04

**Authors:** Ole Petter Rekvig

**Affiliations:** ^1^ Fürst Medical Laboratory, Oslo, Norway; ^2^ Department of Medical Biology, Faculty of Health Sciences, UiT The Arctic University of Norway, Tromsø, Norway

**Keywords:** systemic lupus erythematosus, SLE, SLE classification criteria, unique DNA structures, the causality principle, lupus nephritis, system science

## Abstract

Systemic lupus erythematosus (SLE) is classified by instinctual classification criteria. A valid proclamation is that these formally accepted SLE classification criteria legitimate the syndrome as being difficult to explain and therefore enigmatic. SLE involves scientific problems linked to etiological factors and criteria. Our insufficient understanding of the clinical condition uniformly denoted SLE depends on the still open question of whether SLE is, according to classification criteria, a well-defined one disease entity or represents a variety of overlapping indistinct syndromes. Without rational hypotheses, these problems harm clear definition(s) of the syndrome. Why SLE is not anchored in logic, consequent, downstream interdependent and interactive inflammatory networks may rely on ignored predictive causality principles. Authoritative classification criteria do not reflect consequent causality criteria and do not unify characterization principles such as diagnostic criteria. We need now to reconcile legendary scientific achievements to concretize the delimitation of what SLE really is. Not all classified SLE syndromes are “genuine SLE”; many are theoretically “SLE-like non-SLE” syndromes. In this study, progressive theories imply imperative challenges to reconsider the fundamental impact of “the causality principle”. This may offer us logic classification and diagnostic criteria aimed at identifying concise SLE syndromes as research objects. Can a s*ystems science approach* solve this problem?

## Introduction

*“Insanity is doing the same thing over and over and expecting different results”. Albert Einstein^1^*

This study analyses what we understand and do not understand about systemic lupus erythematosus (SLE) and how we interpret and intellectualize current SLE classification criteria, definitions, and dogmas.

SLE is an enigmatic and consequently inexplicable syndrome, currently authorized by incomprehensible SLE classification criteria (1)—and by the vague and imprecise impact of the causality principle in theoretical and practical contexts. Such problems unveil SLE as a poly-phenotypical and poly-etiological syndrome that contrast—and argue against—the “one disease entity” paradigm [discussed in References (1, 2)]. In this study, research directions will be discussed with the perspective of transforming the enigmatic SLE into a more rational and explicable syndrome. The focus will be on, but not limited to, critical viewpoints on SLE classification criteria, the role and specificity of humoral autoimmunity, and the origin and pathophysiology of lupus nephritis as exemplified in a causality context.

Our insufficient understanding of the clinical condition denoted SLE depends on i) how we operate and interpret classification criteria that categorize SLE cohorts (1); ii) how and why we neglect relevant historical data on unique DNA structures, their immunogenicity, and relevant autoimmunity in rheumatological and clinical immunology contexts (2); and iii) how opposite and contradictory pathophysiological models, as is relevant for lupus nephritis, are left inconclusive and uninvestigated for decades. These points are the frames for the following theoretical discussion.

These problematic topics have provided us with inconsistent SLE cohorts and, consequently, insufficient research perspectives (1). For example, SLE as classified by established criteria may not constitute a “one disease entity” simply because the criteria do not reflect the causality principle that could lead to a more homogenous syndrome. Therefore, classified SLE cohorts may consist of patients that belong to the idiom “a one disease entity”—in other words described as “genuine SLE”. These are promoted by a central etiology. Other patients in an SLE cohort may belong to various groups of “poly-phenotypic SLE” or ultimately belong to a more diffuse group of patients classified by criteria—the “SLE-like non-SLE disorders”. The latter would be in terms of non-SLE diseases that imitate SLE.

In line with these terminologies, classification criteria authorize cohorts of classified SLE patients with or without anti-dsDNA antibodies, with and without lupus nephritis—in general with or without individual classification criteria (1). The clinical pictures of all these patients can be implemented in the same SLE cohort aimed to study etiology, pathogenesis, and, e.g., experimental therapies! Basically, the core question is whether all SLE classification criteria causally belong to—and identify—one dominant origin that promotes a “one disease entity” in harmony with “the causality principle”. Probably, they do not!

In order to transform SLE from being an enigmatic syndrome into a rational and explicable disorder, we have *to re-introduce historical data on DNA structures and their unique auto-immunogenic potentials on one side and focus on links to the causality principle on the other. This means implementing causality to understand the etiology of consequent downstream inflammatory networks—i.e., interactive and interdependent symptoms (defined as criteria) that may explain the nature of SLE*.

## DNA—the life’s most basic and vital macromolecule: why are its unique and dynamic structures ignored in clinical immunology?

Miescher described nuclein in 1871 (3, 4), later named as DNA. In the aftermath of Miescher, scientific studies, experimental data, and biochemical analyses of DNA structures have reached an unconditioned status as central achievements in the history of science. The biochemical and biological impact of DNA belongs today to our most basic and quintessential knowledge related to the inheritance and reproduction of species. During decades after Miescher’s pioneering studies, a considerable amount of data have accumulated on structure–function relationship centered in dynamic DNA configurations—basically linked to gene expression and inheritance but also to their influence on clinical-related autoimmunity [as how immune responses are controlled, activated, and tolerated; reviewed in (2, 5–7)].

Considering the biological impact of DNA structures, it is unexpected and unpredicted to observe that a detailed description of DNA structure–functions as life’s most basic and vital molecular processes is largely ignored in modern autoimmunity, autoimmune pathogenesis, and rheumatology [see, e.g., References (8–10)]. Current mainstream thinking in DNA-associated clinical autoimmunity—and consequent praxis—is based on simplified models and theories that have resulted in dogmatic but invalid hypotheses, questionable and unclear scientific results, and biased interpretations.

If we consider the current literature, DNA in autoimmunity is handled as “*ssDNA*” and as “*dsDNA*”. The latter is clearly described in the central literature on autoimmunity and on SLE classification criteria impacted to classify and diagnose SLE^2^ (8–10). The dynamic transformation of intricate DNA structures, associated with their involvement in gene regulation, gene expression, DNA replication, and DNA repair (11), has not been considered important in contemporary studies on DNA immunogenicity (2). *This provocative presentation is problematic and challenging to contemplate and articulate but necessary to perform.*

### Impact of DNA structures on autoimmunity—a historical account—and a lesson to learn

In 1957, antibodies to DNA in SLE patients were reported in four independent studies (12–15). These reports authenticated the beginning of an intense scientific époque, which culminated in experiments that described molecular and cellular requirements explaining why these anti-DNA antibodies appear (5, 16–21) and why they were claimed unique in SLE as classification and diagnostic criteria (10, 22). The latter statement is mistrusted today because these requirements are universal in a physiological context and are relevant also for the normal immune system [discussed in References (23–25)]. That is, *anti-B DNA^3^ antibodies are not unique to SLE.* This statement harmonizes with the fact that *anti-dsDNA antibodies were described in the context of infections already in 1938–1939* [discussed and reviewed in Reference (23)].

Many inconsistent scientific dogmas have hampered insight relevant to understanding DNA as an immunogen. Early experimental data promoted the concept that *mammalian B DNA* was non-immunogenic while ssDNA and synthetic DNA structures were potent immunogens (26–28). The dogma that settled B DNA as hapten-like and non-immunogenic progressed into a long-lasting principle that only recently became obsolete. While many versions of DNA (ssDNA, synthetic ssDNA/dsDNA, and even the left-handed Z DNA helix) were immunogenic in complex with the immunogenic carrier protein methylated bovine serum albumin [mBSA; see, e.g., References (29, 30)], mammalian B DNA remained indisputably immunologically inert (18, 26). During the 1980s, a confusing and disappointing conclusion emerged: mammalian B DNA was excluded from Sercarz’s hapten-carrier theorem (see below) by classifying B DNA as immunologically inert (26–28).

During the 1990s, this paradigm changed as a consequence of three new experimental concepts. The pioneering experiments in Tony Marion’s laboratory were a breakthrough in this context. He and his coworkers dismissed mBSA as a carrier protein for B DNA in the hapten-carrier paradigm and substituted it with a potent T-cell immunogenic peptide derived from the parasitic euglenoid (31) *Trypanosoma cruzi*—the Fus1 peptide. This artificial complex, B DNA-Fus, induced anti-mammalian B DNA antibodies, and the non-autoimmune experimental animals developed lupus-like nephritis^4^ (19, 32). This experiment teared down the dogma saying that mammalian B DNA was immunologically inert!

A similar *in vivo* model was developed in Rekvig/Moens laboratories. They introduced polyomavirus T antigen-expressing plasmids in mice and revealed that expressed T antigen associated with chromatin in T antigen-expressing dying cells, formed stable complexes, and induced antibodies to mammalian B DNA, histones, and transcription factors (20, 21, 33). *Thus, the chromatin–T antigen complex stimulated B cells theoretically specific for any accessible ligand contained in the chromatin complex provided they presented (in this example) the complexed T antigen peptides to cognate helper T cells* (20, 21, 33).

At the same time, Pisetsky et al. described the production of anti-bacterial DNA antibodies in normal mice in response to bacterial CpG-rich DNA-mBSA complex immunization and made an observation with key importance; the immunization protocol in pre-autoimmune mice accelerated production of potential pathogenic anti-mammalian B DNA (30, 34, 35). This implied that a ubiquitous source of immunogenic dsDNA, the bacteria, could induce autoimmunity to mammalian B DNA in pre-autoimmune animals. *This brought infections as a central immunogenic source for anti-dsDNA autoimmunity into the SLE-related autoimmune forum*. This paradigm shift is clinically important when we consider the predisposition of infections in SLE [principally discussed in References (22, 23)].

These results have clearly communicated to us the clinical impact of chromatin autoimmunity in diagnostic and pathogenic contexts. The data expand the impact of DNA from being a gene “bank” to, in a wider perspective, expanding our understanding of SLE and its contextual autoimmunity. From such data, chromatin autoimmunity as a principle has taught us basic regulatory principles of the immune system [reviewed and discussed in References (5, 7, 23, 36, 37)]. These progressive experimental studies inform us about the importance of new scientific research directions related to the autoimmune pathogenesis of lupus nephritis (25), *and they taught us the lesson not to adhere to scientifically non-productive dogmas (see citation of Albert Einstein above)*.

### Anti-dsDNA antibodies as a potential biomarker for SLE: how do we define anti-dsDNA antibodies correctly, which conditions are they linked to, and why are scientific hypotheses complicating their clinical impact?

*The functional Sercarz’s hapten-carrier theorem for induction of anti-DNA antibodies* (27, 28, 38) *inherits a perpetual and self-explaining dogma: the anti-dsDNA antibodies cannot be specific for SLE*. This statement is explained as follows: all people embody reactive DNA-specific B cells (39–41). They are not tolerant—rather, they are promiscuously responsive (42, 43). *Helper T cells* are tolerant of chromatin-associated autologous proteins (23). They can therefore not provide cognate T-cell help for DNA-specific B cells! However, in the context of, e.g. viral infections, DNA-binding viral proteins may associate with chromatin, thus creating a functional dsDNA (hapten-like) virus-derived (carrier–) protein complex. Since we all inherit DNA- and virus protein-responsive B and T cells, this hapten-carrier version explains why anti-dsDNA antibodies are not—and cannot—be specific for SLE (23).

Over decades, numerous studies have been published that attempted to describe the unique clinical impact of anti-B DNA antibodies in SLE. Surprisingly, most of these studies canceled or ignored experimental (19–21, 30, 35) and observational data that demonstrated the production of anti-dsDNA antibodies in the context of infections and malignancies [(23, 44) reviewed in Reference (23)] and sporadically in other conditions (45).

### Ignored legendary data on DNA structures with potential impact on the specificity of DNA immunogenicity

One central but disregarded science-based fact diminishes the impact of DNA in autoimmune pathogenicity and disease associations: the existence of disparate DNA structures, like ssDNA, bent dsDNA, elongated dsDNA, Z DNA, cruciform DNA, and bacterial and viral DNA, with theoretically unique and unrelated immunogenic potentials. This may raise critical questions around the clinical impact of “the anti-dsDNA antibody” and whether this specificity may remain “unique” for SLE [(8–10), discussed in References (2, 46)]. The definitive documentation of a clear immunogenic potential of any of the DNA structures alters this view [discussed in References (22, 35, 44, 47)]. The arguments in this study hint at why anti-dsDNA antibodies appear in SLE *among many syndromes* predisposed to conditions like infections and malignancies (23, 44, 48–53).

The latter observations basically confront the motivation for putting weight on anti-dsDNA antibodies in SLE classification criterion sets (8–10). It is obviously important to include non-SLE patients disposed for infections and malignancies as corresponding control groups.

Therefore, the term “anti-dsDNA antibody” [as in SLE classification criteria (8–10)] is a simplification that conceals the impact of antibodies on disparate, unique, and functional DNA structures.

## Theoretical impact of unique DNA structures as individual immunogens

Since the early 1870s, DNA structures and their biological operations have been investigated and are still a prevailing and central research focus [reviewed in Reference (2)]. These studies have identified a series of structurally unique DNA configurations that must be brought to this discussion forum. These unique DNA structures exert distinctive roles in gene biology and biochemistry but are still ignored in auto-immunological contexts.

### DNA: structure diversity and structure–function relationships—a concise narrative

Miescher et al. studied in the 1870s the nuclear substance nuclein and suggested that nuclein could have something to do with inheritance, and later, he theorized that a hereditary alphabet based on stereochemistry was hidden in the nuclein. This could explain how *variation* is generated [discussed in Lamm et al. (54)]. Mostly, Mischer’s concept of DNA as a carrier structure for inheritance has been ignored in this narrative (54). In 1909, the identification of the chemical nature and composition of DNA was described by Levene et al. (55). They determined that DNA was composed of the central nucleobases adenine, guanine, thymine, and cytosine.

Important scientific results on DNA structure emerged from central studies of Erwin Chargaff and Rosalind Franklin. Chargaff et al. (56, 57) defined the two Chargaff rules on the composition of double-stranded DNA, saying that the number of guanine equals the number of cytosine and that the number of adenine equals the number of thymine in all species [reviewed and discussed in Reference (2)]. In 1952, Franklin produced pioneering high-resolution photographs of crystallized DNA fibers and interpreted from these pictures that DNA was a double-stranded helical structure (58–60). This model harmonized with the Chargaff’s rules. Franklin and Chargaff were therefore the first to describe the basic structure of DNA as a double-stranded helix.

**Figure 1:**
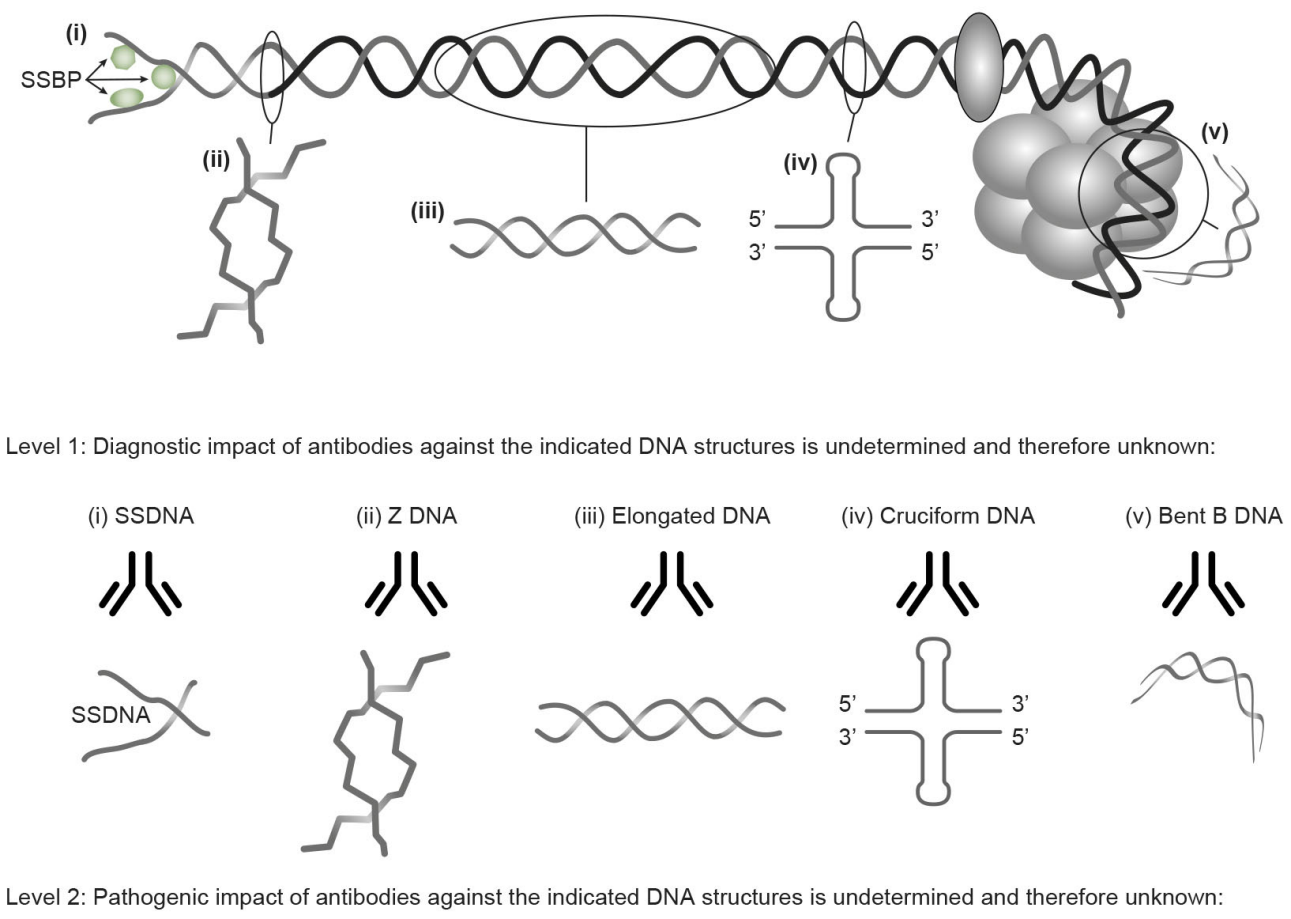
Transcriptionally active DNA expresses distinct DNA structures—each structure is a unique antigen. (i) The B DNA helix is opened by single-stranded binding proteins (SSBPs; which stabilize ssDNA and polymerize, which are involved in replication and repair). (ii) Z DNA is a left-handed, high-energy, double helix. Z DNA forms during transcription as a result of torsional strains that depend on interaction with mobile polymerases. Z DNA is associated with linker DNA. (iii) Elongated (linker) DNA is a relaxed and stable, right-handed, low-energy form of B DNA. Cruciform DNA is another dsDNA structure (iv) and is different from B and Z DNA. Its formation requires that sequences (palindromes) in one strand are repeated on the other strand in opposite directions. The cruciform structures are, like Z DNA, higher-energy structures. Compacted B DNA as in core nucleosomes is defined as bent B DNA (v). Bent DNA is a compacted structure influenced by the histone octamer and histone H1. These structures (i–iv) are unique in terms of inducing highly specific antibodies with potential pathogenic impact if chromatin fragments are exposed *in situ* (see **Figure 2**). This figure demonstrates the unique immunogenic DNA structures [revised from Reference (2)].

Essential in this context is that Franklin and Goslin were the first to describe two unique forms of DNA beyond its archetypical double-helix structure: the A and B DNA (58). The B DNA was later described as a dynamic fluctuating bi-structural DNA shape: elongated (61, 62) or bent dsDNA (63, 64). SsDNA (65, 66), Z DNA (26), cruciform DNA (67), and other structures were all *described in the context of specific functions of DNA* [see below, **Figure 1**, and References (2, 68, 69)]. Watson and Crick (70, 71) combined data from Chargaff’s studies and Franklin’s X-ray data and finally confirmed and settled the helical structure of DNA in which A pairs with T, and C pairs with G (corresponding to Chargaff’s rules).

**Figure 2:**
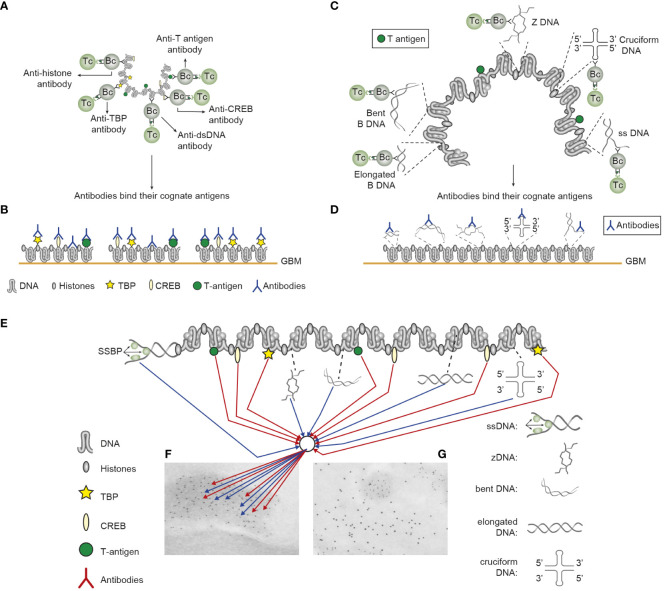
Induction of anti-chromatin antibodies **(A)** and anti-DNA structure-specific antibodies **(B)**—demonstration of Sercarz’s hapten-carrier theorem involving expression of polyomavirus T antigen as immunogenic carrier protein. **(A)** Injection of non-autoimmune mice with plasmids expressing polyomavirus DNA-binding T antigen induces production of antibodies to DNA, T antigen, mammalian histones, and certain transcription factors like TATA-binding protein (TBP) and cAMP-responsive element-binding protein (CREB) by cognate interaction between chromatin specific B cells and polyomavirus T antigen peptide-specific helper T cells. **(B)** These antibodies bind chromatin-antigens exposed in GBM and promote nephritis. **(C)** Identical immunization regimes induce autoantibodies against elongated B DNA, bent B DNA, Z DNA, cruciform DNA, and ssDNA. **(D)** The anti-DNA structure-specific antibodies promote nephritis by binding exposed DNA antigens in GBM. Autoimmune B cells and operational immune T cells cooperate in this model. **(E)** All the induced anti-chromatin antibodies have pathogenic potentials if binding exposed chromatin, as is demonstrated in GBM as electron-dense structures (EDS in panels **F**, **G**). The induced autoantibodies (stained with 5-nm gold particles; **F**) bind chromatin fragments. Chromatin fragments are surrounded by GBM structures that bind anti-laminin antibodies added to the section *in vitro* (10-nm gold particles; **G**). **(A–D)** Modified from Reference (25).

Later, studies of DNA structure–function relationships *in chromatin* were performed by Olins and Olins (72), Kornberg et al. (73, 74), Richmond et al. and Luger et al. (75, 76), Laskey et al. (77), and others. They contributed to our understanding of the consequent interdependency of DNA structures and functions^5^ and the biology of chromatin-associated regulatory and chromatin compaction proteins—and a new view on concise DNA structure immunity (2).

### Efferent immunogenic impact of DNA structures

DNA embodies all aspects of inheritance necessary to maintain the life and integrity of a species (69, 78–80).

Notably, the manifold of DNA structures (**Figure 1**) preserves a striking, still largely ignored relevance in an autoimmune context: *each structure has a unique ability to induce highly specific anti-DNA antibodies within experimental and autoimmune frameworks* (2).

Thus, from published studies over more than seven decades, it is clear that DNA structures and their functions tell a quite different story than the narrative about the sole existence of “the anti-DNA antibody” (2). This history contrasts general clinical viewpoints about anti-dsDNA antibodies operating as a single antibody specificity reacting with “the dsDNA” (8–10, 81–83). This somewhat naïve paradigm is not in agreement with the fact that each DNA structure has the potential to induce highly segregated and structure-specific anti-DNA antibodies [**Figure 1** (2)].

## The imprecise term “anti-dsDNA antibody” comprises disparate unique specificities: these are not collectively detected by any single assay principle, and their clinical impact is not investigated

“The anti-dsDNA antibody” holds an archaic position in SLE as a unique classification and diagnostic criterion. In light of what we know today, this position is principally wrong, *as studies of disease association(s) of anti-dsDNA antibodies have been conducted without rational references to assay principles designed to detect individual DNA structure specificities* (2).

### Segregated anti-structural DNA antibody specificities—an aspect that principally undermines the existing clinical authority of “the anti-dsDNA antibody” as a classification and diagnostic criterion

Why do anti-dsDNA antibodies exist as a unique classification criterion in SLE (22)? During the decades following the 1957 discoveries of anti-DNA antibodies in SLE, two important aspects of the anti-B DNA antibody were comprehended: i) the promiscuous cross-reactive nature of anti-dsDNA antibodies (42, 84–89) and ii) the non-immunogenic nature of B DNA.

Cross-reactivity precipitated the dogma declaring that the existence of anti-B DNA antibodies depended on cross-stimulation by heterogeneous non-dsDNA structures, like phospholipids, different polypeptides, and non-mammalian bacterial DNA (23, 35, 42, 90–94).

If B cells were stimulated by a peptide, they were in immunological terms subjected to somatic hyper-mutations—affinity maturation—and the antibody-specificity converges toward the peptide immunogen. However, the clones were initially specific also for cross-stimulated dsDNA. Will these be further expanded by the proclaimed non-immunogenic, exposed chromatin-associated dsDNA, or will these DNA-specific clones passively expire? In other words, will cross-stimulated clonal anti-B DNA antibody responses be transient if occurring on a normal, non-autoimmune background, *or can a peptide progressively expand and affinity-maturate DNA-specific clones?* These concise questions remain unanswered.

### Outdated algorithms: new problems, new challenges

The radical paradigm shifts described above confirmed that B DNA is immunogenic, consistent with Sercarz’s hapten-carrier theorem. This, however, created some new intellectual problems: in view of the verifiable immunogenic potential of B DNA independent from an autoimmune background, are anti-dsDNA antibodies still specific for SLE? Do disparate anti-DNA structure-specific antibodies reflect the same clinical impact as the dogmatic anti-B DNA antibody? Are DNA or membrane ligands targeted by anti-dsDNA antibodies in lupus nephritis? Finally, why are theoretical, observational, and experimental scientific studies, basically aimed to understand SLE, ignoring i) classical and legendary data on DNA structures, ii) conflicting interpretations on lupus nephritis pathogenesis (see below), and iii) “the causality principle” in the context of the SLE classification criterion selection process. Problematically, these criteria promote today’s inconsistent SLE research strategies applied to potentially illogical SLE cohorts [discussed in Reference (1)].

In sum, it is now settled that DNA is not one “structurally dead” and inflexible macro-molecule. The dynamic and changing DNA structures are caused by histone mobility, enzymes, and regulatory proteins [reviewed by Zhang et al. (95)]. This means that over 7 decades of provokingly reflective and insightful high-impact research, DNA structures are well acquainted with relevant basic scientific disciplines linked to genetics, biochemistry, and regulation of gene transcription.

These scientific complications imply, so far, several consequences for SLE research: the insight into manifold DNA structures has expanded from an archetypical double helix structure into several concise and specialized function-related DNA structures (95). We must therefore bring to the discussion forum a concise proposition: *the B DNA is immunogenic*. *Consequently, this relates also to unique DNA structures according to Sercarz’s theorem—they may all possess an immunogenic potential* (2). This statement predicts the *de facto* existence of several concrete and unique anti-structural DNA antibody specificities (2). Notably, their individual immunopathogenic and clinical impacts have not been subjected to research in clinical hypothesis-driven contexts. We definitively need a more profound insight into assay principles that detect each of the clinically existing anti-structural DNA antibodies.

This leads to several new hypotheses:

1. Since B DNA is a weak immunogen (26), while, e.g., ssDNA or Z DNA is an effective immunogen (26, 39, 96), every structural DNA-specific antibody may have a unique impact as diagnostic or classification criterion, as is currently and historically relevant for the anti-mammalian B DNA antibody (8–10). The logic hypothesis imposed from this information could be that the easier the DNA structures promote immune responses, the less specific for a disorder will the emerging antibodies be. For example, immunity to ssDNA and Z DNA may inherit a lower diagnostic impact than, for example, antibodies against the bent mammalian dsDNA because of the relatively poor immunogenicity of bent dsDNA (2, 23).
2. Since anti-DNA antibodies are induced by DNA structures as those presented in chromatin, the produced antibodies must be able to bind the same cognate DNA structures when they are exposed as chromatin fragments in lupus nephritis. This must consequently mean that they are all potentially pathogenic, with one reservation: that the density of each target structure is sufficient in chromatin-associated DNA to bind adequate amounts of IgG anti-DNA structure-specific antibodies to mediate Fc gamma-dependent complement-activating inflammatory processes.
3. Importantly, the autoimmune responses as presented in **Figures 1**, **2** represent arguments against cross-reactivity of the disparate anti-dsDNA antibodies with membrane and matrix ligands to explain antibody-dependent lupus nephritis (see **Figure 2**). This whole repertoire of anti-chromatin antibodies may be involved in nephritis through cognate interaction with exposed chromatin ligands [**Figure 2** (25, 97)] and not with cross-recognized membrane antigens. It is unlikely that all of a large group of anti-chromatin antibodies will, by chance, cross-react with a small number of membrane ligands (25, 53). Therefore, anti-chromatin antibodies are pathogenic if they bind exposed chromatin fragments *in situ* (see details discussed below).
4. In the absence of extracellular chromatin-associated DNA, an alternative inducer of anti-DNA antibodies may be mitochondrial DNA (Mit DNA) (98). Mit DNA has been reported to be involved in innate immunity (99) and inflammatory processes (98, 100, 101). Anti-Mit DNA antibodies are involved in disease processes (99). There are few studies that report the cross-reactivity of anti-nuclear antibodies with Mit DNA (101). Thus, whether anti-Mit DNA antibodies cross-react with exposed nuclear DNA is not firmly established. However, if Mit DNA is exposed in tissue, anti-Mit DNA antibodies may theoretically express pathogenic effects by binding Mit DNA *in situ*. This is, however, a speculation and is not based on experiments or descriptive analyses. In contrast, both viral DNA (102–105) and bacterial DNA (30, 34) have the potential to induce mammalian nuclear anti-dsDNA antibodies similar to such specificities in SLE (22, 23, 25).

To describe the origin of anti-dsDNA antibodies, the most probable explanation is founded on the release of (nuclear) chromatin as a result of inflammatory-mediated silencing of renal DNase 1 gene and loss of DNase 1 endonuclease activity. This may explain the efferent and afferent phases of immune response to nuclear DNA and may explain the basic processes in the chromatin model (see below—the “chromatin model” for induction of pathogenic anti-chromatin antibodies is discussed).

## Contemporary hypotheses related to the pathogenesis of lupus nephritis: what defines pathogenic anti-chromatin autoantibodies—and are they all *always* pathogenic?

*Anti-dsDNA antibodies are pathogenic in SLE.* However, this statement is controversial, irrespective of whether alone or in combination with other anti-chromatin antibodies (**Figure 2**). What we need to comprehend from an enormous amount of studies (106) is to understand what makes the anti-DNA/anti-chromatin antibodies pathogenic—*and in which contexts*.

### Clinical impact of anti-structural DNA specific antibodies: are they pathogenic and diagnostic factors in SLE—and what have we lost along the scientific avenue?

The archaic “anti-dsDNA antibody” terminology is founded on an outmoded comprehension in historical as well as contemporary contexts. Today, we may, in harmony with the authoritative attribution rules for SLE classification criteria (8–10), infer that *any structure-specific anti-DNA antibody, detected in any anti-DNA antibody assay using any target dsDNA molecule, is valid as a criterion for SLE, as long as the target DNA is dsDNA* (8–10).

We may formulate a simple and open-minded hypothesis: *the more readily an antibody is induced, the less specific is the antibody for SLE.* Any anti-dsDNA antibody may appear in different SLE/non-SLE conditions (2), while those DNA structures that are more effectively controlled by tolerance may induce antibodies with a closer connection to SLE ()?. In other words, antibodies against, e.g., ssDNA or Z DNA, may be less specific for SLE than anti-bent B DNA antibodies, as the former structures are more effective immunogens than the latter (2, 26, 107). This hypothesis remains open and still un-investigated.

### Anti-dsDNA antibodies: what makes them nephritogenic—specificity for chromatin-associated DNA or inherent GBM structures—a discussion relevant for most anti-chromatin auto-antibodies

The anti-dsDNA antibodies may inherit a nephritogenic potential by two incommensurable characteristics: their specificity for exposed chromatin-associated dsDNA, *the chromatin model*, or their cross-reactivity for inherent matrix/membrane ligands, *the cross-reactive model*.

In this section, the nephritogenic impact of anti-DNA/anti-chromatin antibodies (**Figure 2**) will be discussed. These antibodies may be specific for dsDNA without further definition [as incurred in the SLE classification criteria (8, 9, 82)], or for chromatin-associated dsDNA originated from apoptosis or neutrophil extracellular traps (NETs) (108–110), in addition to specificity for chromatin-associated proteins. Alternatively, they may cross-react with inherent membrane structures [see, e.g (23, 25, 42, 94, 111–115) and Table 2 in Reference (25)]. This dilemma is not comparatively investigated in terms of *systems science* (116) involving different congruent and corresponding scientific methods based on focused hypotheses.

*Working hypothesis*: Today, we are principally not able to unequivocally explain the nephritic process at a molecular level, although some detailed studies indicate relevance for the chromatin model (25, 53, 117–119).

### Lupus nephritis: the chromatin model

Anti-chromatin antibodies may bind extracellular chromatin fragments exposed in glomeruli and form immune complexes—either *in situ* or trapped from circulation. These may be detected early and primarily be detected in the mesangial matrix (120, 121) and promote clinically mild or silent mesangial nephritis. This condition can be diagnosed by a combination of modest proteinuria and deposition of co-localized chromatin fragments and IgG antibodies in the mesangial matrix (120, 121). These IgG antibodies comprise specificities for dsDNA, histones, and transcription factors (20, 122–125). The model is consistent with a Type III immune complex-mediated inflammation [(126); see a model illustrated in **Figure 2**]. Mesangial nephritis promotes the progression of lupus nephritis into end-stage organ disease. This type of nephritis is characterized by heavy deposits of chromatin–IgG complexes in the Glomerulus basement membrane (GBM) [discussed in References (25, 53)].

The chromatin model is complex and evidently involves a wide spectrum of chromatin-specific antibodies. The model (illustrated in **Figures 2A, C**) exemplifies Sercarz’s hapten-carrier theorem: all B cells specific for chromatin-associated ligands (e.g., DNA, histones, non-histone regulatory proteins, and transcription factors) internalize in this model the immunogenic chromatin-associated polyomavirus T antigen (or auto-immunogenic histones) and present T antigen (or histone) peptides to non-tolerant helper T cells in context of HLA class II molecules (20, 21, 127). The net result is the production and binding of cognate chromatin-specific autoantibodies (see details in **Figures 2B, D, E-G**). Collectively, most of the various anti-chromatin antibodies have not been seriously considered as inducers of lupus nephritis, except for “the anti-dsDNA antibody” [as described in References (8–10)].

All anti-dsDNA antibodies (except anti-cruciform antibodies, which are not analyzed yet in clinical contexts) are induced in SLE or are experimentally inducible *in vivo* (2, 23). The chromatin structures (DNA and proteins) must therefore have been accessible to highly specific B-cell antigen receptors (2, 20). *Then, it is likely that the antibodies access and recognize the same universe of DNA structures and chromatin-associated proteins when chromatin fragments are exposed in, e.g., glomeruli* (**Figures 2A–G**).

It is in this context that it is unlikely that the whole spectrum of distinct anti-chromatin antibodies collectively cross-react with few inherent glomerulus matrix/membrane constituents.

### Lupus nephritis: the cross-reactive model

In contrast to the cognate interaction of antibodies with dsDNA, a growing number of studies have demonstrated that anti-DNA antibodies may cross-react with glomerular matrix and membrane ligands, e.g., laminin, entactin, or collagen (25, 117). This pattern of antibody recognition gradually created a new paradigm: the anti-dsDNA antibodies promote nephritis by binding inherent GBM structures *in vivo* (86, 93, 112, 128–133), i.e., a model compatible with a Type II antibody-dependent inflammation (126).

The dual specificity of nephritogenic antibodies for dsDNA and non-dsDNA membrane ligands does not clarify which of the antigens are targeted *in vivo*.

This problem further escalated into an intellectual conflict that still is not investigated in comparative studies, is not resolved, and is currently largely ignored as a problem! Today, there is no visible research program on the horizon that may solve the problem of whether, and how, cross-reacting anti-dsDNA antibodies are involved in lupus nephritis (see a concise hypothesis-based discussion in the next section).

### A comparative study of “the chromatin” and the “cross-reacting” models: a focus on their dynamic and progressive natures, and structure and composition of immune deposits in glomeruli

How do the chromatin and the cross-reacting models comply with the clinical course of lupus nephritis? Implementation of the causality principle must in this context be considered.

**Figure 3:**
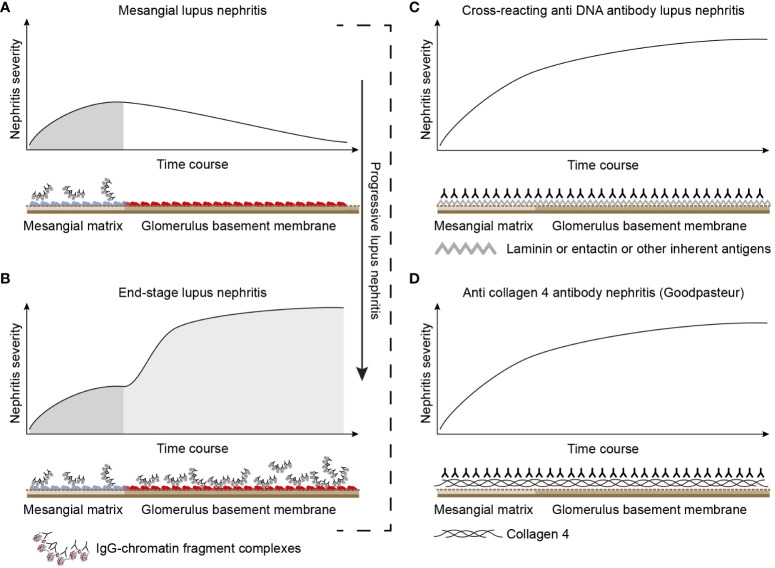
Theoretical disease profiles differ due to the molecular specificities of the autoantibodies. **(A)** Anti-dsDNA antibodies form immune complexes with assumed circulating small chromatin fragments that accumulate in glomerulus mesangium (121). This promotes mild, early mono-phasic, and transient lupus nephritis (Phase 1). Under certain conditions, mesangial inflammation promotes silencing of the renal DNase 1 endonuclease (121). In reflection of loss of DNase 1 enzyme activity, chromatin released from dead cells accumulates as undigested chromatin fragments in complex with anti-chromatin antibodies in mesangial matrix and in GBM [see **Figure 2** (121)]. This promotes end-stage nephritis (**B**; Phase 2)—*a second-phased progression of lupus nephritis.* This model contrasts with the cross-reacting model (described in panel **C**). Here, a cross-reacting anti-DNA antibody binds inherent membrane antigens (like laminin, collagen, and entactin) shared between the mesangial matrix and GBM. Therefore, the nephritis profile is mono-phasic, as the mesangium and GBM are simultaneously affected by antibodies. This mono-phasic nephritis is complementary to nephritis in Goodpasture syndrome **(D)**. Goodpasture-type nephritis is caused by anti-collagen IV antibodies that bind collagen structures shared by the mesangial matrix and the GBM. The antibodies therefore promote a mono-phasic profile of the nephritis in cross-reacting lupus nephritis models and Goodpasture syndrome.

*The chromatin model* is principally characterized by a progressive profile that implements two immunopathological aspects: Phase 1 and Phase 2 (**Figures 3A, B**). *Phase 1* is caused by an early and modest production of anti-dsDNA/anti-chromatin antibodies (117, 120, 121, 134). The pure presence of the anti-dsDNA antibody causes accumulation of immune complexes in the mesangial matrix—observed by immune electron microscopy as electron-dense structures [EDSs; consisting of chromatin–IgG complexes (121)] and clinically silent or mild mesangial nephritis with low-graded proteinuria (**Figure 3A**).

**Figure 4:**
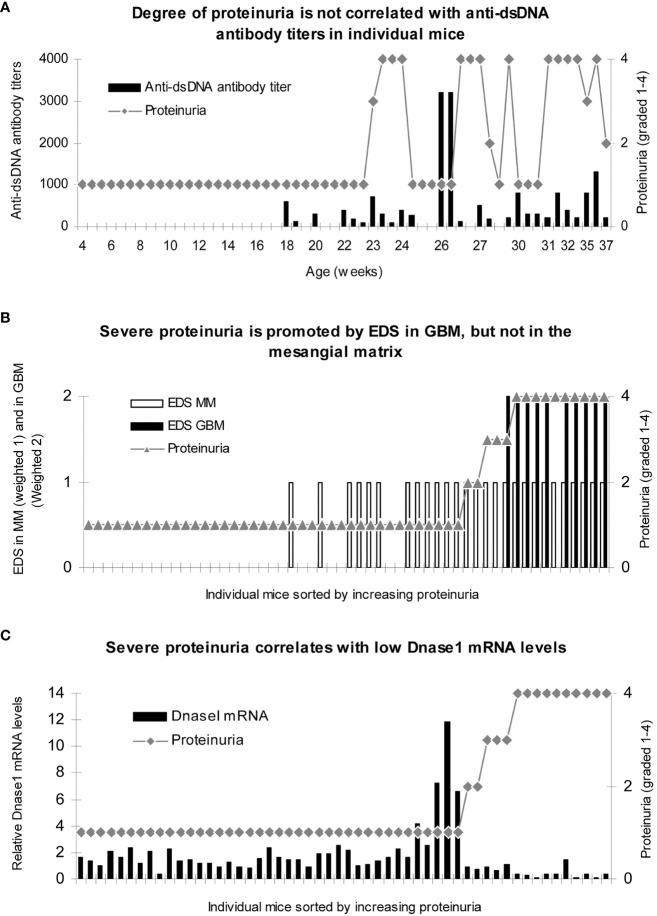
Severe proteinuria correlates positively with chromatin fragment deposits [observed as electron-dense structures (EDS) in GBM] and inversely with renal Dnase 1 mRNA levels. Renal tissue was collected from female (NZBxNZW)F1 (BW) and female age-matched BALB/c mice (Jackson Laboratory, Bar Harbor, ME, USA) sacrificed approximately every second week (n   = 3) from the age of 4 weeks until development of end-stage disease in the BW mice, clinically defined when severe proteinuria developed (≥20 g/L). Tissue was snap-frozen for protein extraction, preserved according to Tokuyasu for immune electron microscopy (138), or preserved in RNAlater (Ambion Inc., Austin, TX, USA) for mRNA analyses. Serum and urine samples were collected at 2–3-week intervals and stored at −80°C. No correlation between degree of proteinuria and levels of anti-dsDNA antibody titers was observed **(A)**. To analyze if loci for electron-dense structure (EDS) deposits had impact on proteinuria, data on proteinuria and presence of EDS in the mesangial matrix (weighted 1 in B) or in the GBM (weighted 2 to make a visual distinction from mesangial deposits) were combined for each mouse and sorted by ascending values of proteinuria. Severe proteinuria (≥20 g/L) was, except for one mouse with intermediate proteinuria (≤3 g/L), exclusively observed in mice with EDS in GBM **(B)**, while intermediate or mild proteinuria was observed in mice with mesangial matrix deposits only **(B)**. In panel C, the degree of proteinuria and renal Dnase 1 mRNA levels were paired and sorted by ascending proteinuria levels. Severe proteinuria (≥20 g/L) correlated with a substantial loss of Dnase 1 mRNA (and enzyme activity; **(C)**. Thus, in mice selected for proteinuria ≥20 g/L, renal Dnase 1 mRNA was nearby lost in all mice but one **(C)**, and deposits of chromatin–IgG complexes (observed as EDS) in GBM were observed only in these mice. This instructive figure is copied from Reference (121). For statistical analyses, see Table 1 in Reference (121).

These mesangial immune complexes may incite a local inflammation that is associated with an abrupt silencing of the renal DNase 1 gene, the dominant renal endonuclease (109, 135). This is consistent with increased glomerular accumulation of undigested extracellular chromatin fragments in complex with IgG anti-dsDNA antibodies (108, 121, 123, 135–137) in both the mesangial matrix and GBM (Phase 2, **Figure 3B**). This pattern reflects the progression of lupus nephritis (and severe proteinuria) into end-stage organ disease (121, 137). The chromatin model linked to progressive loss of renal DNase 1 explains *Phase 2* of the bi-phasic progressive lupus nephritis (see details in **Figures 3B**, **4**).

In **Figure 4A**, it is demonstrated that the degree of proteinuria in mesangial as well as in end-stage lupus nephritis does not correlate with fluctuations in anti-dsDNA antibody titers. In contrast, the progression of proteinuria correlated significantly with the progressive accumulation of EDS [representing chromatin–IgG complexes (25, 120, 121)] in the mesangial matrix and subsequently in GBM (**Figure 4B**). A striking inverse correlation was observed between the degree of proteinuria and loss of renal DNase 1 gene expression (**Figure 4C**). Similar murine characteristics of progressive lupus nephritis are observed in human SLE (25, 108, 136, 139).

*The cross-reacting lupus nephritis model* is characterized by a quite different dynamic profile (**Figure 3C**). This is basically determined by the equal distribution of the target membrane ligands for the antibodies in the mesangial matrix and the GBM [reviewed in References (25, 53)]. The target molecules, such as laminin, collagen, or entactin, are shared by membranes in glomeruli and alveoli on one side (140) and by glomerular matrix and membranes on the other (141–144). This opens for the intriguing concept that the cross-reacting lupus nephritis model (if true) may principally represent a mirror image of the autoimmune Goodpasture syndrome (indicated and compared in **Figure 3C**, the cross-reacting model, and **Figure 3D**, Goodpasture nephritis). The two latter pathogenic situations are principally similar, as the target antigens in both situations are shared between the glomerular mesangial matrix and basement membranes (145–147).

In a corresponding argumentation, the glomerular immunofluorescence pattern of IgG deposits in Goodpasture-type nephritis is continuous and linear, as would be expected also for the pattern promoted by the cross-reacting model. However, in the chromatin model, the deposits in the mesangial matrix and GBM are granular and discontinuous (121). These differences account for two alternative aspects of lupus nephritis: the mono-phasic nephritis caused by cross-reacting anti-dsDNA antibodies and the bi-phasic nephritis caused by immune complex deposition as predicted—and observed—for the chromatin model (**Figures 2**, **3**). Data so far on lupus nephritis are compatible with the chromatin model (25).

A third distinction between the chromatin and the cross-reacting models relies on the term “cross-reaction” itself. If we dissect the chromatin model, it is complex, as a manifold of anti-chromatin antibodies is produced in SLE [**Figure 2** (23, 148)]. Many of these antibodies bind in, and are eluted from, nephritic kidneys ( (123–125, 149, 150). This fact has one implication: the anti-chromatin antibodies access chromatin-associated nucleic acids and proteins in chromatin structures exposed in glomeruli. Ergo, the B-cell antigen receptor, as well as anti-chromatin antibodies, bind the same accessible antigens in chromatin fragments (151–153).

The manifold of potentially nephritogenic anti-chromatin antibodies (**Figure 2**) is *per se* a clear argument against the cross-reacting model. It is unlikely that the high number of potentially nephritogenic anti-chromatin antibodies together, by chance, cross-react with the relatively few glomerular matrix and membrane proteins. This does not, however, exclude that single, individual anti-chromatin antibodies may contribute to nephritis by cross-binding membrane/matrix constituents, although the majority of the antibodies distinctly cause a Phase 1 nephritis and a subsequent Phase 2 nephritis according to the chromatin model (see **Figures 3A, B**). *This causation cannot be explained by the cross-reacting model.*

The conflicting arguments favoring Type II or Type III nephritis derive from studies over decades, preliminary conclusions, and a lack of international consensus [see, e.g., contrasting viewpoints in References (25, 97, 119, 131–133, 154–156)]. In light of this, it is strange to observe that systematic comparative studies have not been prioritized and are still awaited in the context of the *systems science* algorithm (157). We still need definitive experimental and descriptive evidence for either of the two models, although arguments favoring the chromatin model are relatively strong.

## Concluding remarks

Clinical-related research on dsDNA/chromatin autoimmunity is undisputedly one of the most prominent disciplines in the SLE research field. These research efforts are basically resulting in confusing and inconclusive data when our focus is concerned with diagnostic, as well as pathogenic implications of autoimmunity. We still, after all these years, misinterpret the diagnostic impact of anti-dsDNA antibodies (they are not unique to SLE), and we still do not agree on what these antibodies bind in glomeruli (2), whether dsDNA (as in chromatin) or intrinsic membrane ligands.

We have to rethink, and critically develop, new strategies to gain relevant insight into the delimitation of SLE classification criteria. Are they all causally related to SLE (1)? Are we able to describe pathogenic mechanisms at a molecular level that are operational in SLE (1)? The basis for our contemporary investigations relies on classification criteria that are based i) on criteria that are established by the same procedures today as in 1971 (8–10, 81–83), except that immunological parameters were not implemented in 1971 (8–10, 81–83); ii) the majority of criteria are reiterated over the last 50 years (158); iii) the causality principle has not been implemented in the classification criteria (158); and iv) there are modest explanations that link these dogmatic criteria to SLE as “a one disease entity”. These problems incarnate Albert Schweitzer’s “insanity dogma” cited in the Introduction of this study.

Indeed, we have to develop new hypotheses and research strategies to develop a causality principle-based armamentarium of parameters that is (relatively)? specific for SLE as a “one disease entity”. Basically, we need strategies to separate SLE categories, as like a “one disease entity”, or polyphenotypic and poly-etiological SLE, and to identify disorders that mimic SLE: the “SLE-like non-SLE disorders”—the latter in terms of non-SLE diseases that imitate SLE (1, 158). *Today’s SLE classification criteria do not have logical and understandable rules to distinguish these alternative SLE definitions from each other* (158).

A systems science approach^6^ may provide solutions to solve the basic problems discussed in this study. Basically, systems science brings together various science disciplines and scientists relevant to an actual, often fundamental, problem (116, 157). This means that all aspects are approached to *identify and investigate* the complexity of a problem. The causality principle must be a central scientific element. This means that scientists representing diverse, complementary disciplines must come together, agree to—and define—the problem in order to implement systems science principles to describe the complexity of the problem—and logically to solve it.

## Data availability statement

The original contributions presented in the study are included in the article/supplementary material. Further inquiries can be directed to the corresponding author.

## Author contributions

OR: Conceptualization, Data curation, Formal analysis, Investigation, Methodology, Project administration, Resources, Software, Validation, Visualization, Writing – original draft, Writing – review & editing.

## Footnotes

1 This is a citation repeatedly given by Albert Einstein. The reason for presenting this citation in the present context will be clear while reading this manuscript.

2 All patients that are classified as SLE are enrolled in an SLE cohort. Ergo, they are both classified and diagnosed as having SLE.

3 Throughout this study, B DNA refers to mammalian dsDNA, while dsDNA is used for dsDNA in any species.

4 Is it time to revise the term “lupus-like nephritis”? In many ways, nephritis in, e.g., (NZBxNZW) F1 mice is pathogenetically similar to classes of human SLE (25).

5 DNA in this context not only is various double-stranded DNA versions but also included functional single-stranded DNA. It is therefore correct to use the term “DNA” here without further distinction.

6 Systems science is an interdisciplinary field that studies the complexity of systems in nature, social, or any other scientific field. Some of the systems science methodologies include systems dynamics modeling, agent-based modeling, microsimulation, and Big Data Techniques. Systems science thinking can help researchers understand the factors influencing the distribution and determinants of health and disease in populations by providing information on the broad picture of how individuals and other components of populations are interconnected in society in scenarios in which researchers cannot control the environment (Columbia University Irving Medical Center).
